# Genome Data Uncover Conservation Status, Historical Relatedness and Candidate Genes Under Selection in Chinese Indigenous Pigs in the Taihu Lake Region

**DOI:** 10.3389/fgene.2020.00591

**Published:** 2020-06-09

**Authors:** Chenxi Liu, Pinghua Li, Wuduo Zhou, Xiang Ma, Xiaopeng Wang, Yan Xu, Nengjing Jiang, Moran Zhao, Tianwei Zhou, Yanzhen Yin, Jun Ren, Ruihua Huang

**Affiliations:** ^1^Institute of Swine Science, Nanjing Agricultural University, Nanjing, China; ^2^Huaian Academy, Nanjing Agricultural University, Huaian, China; ^3^College of Animal Science, South China Agricultural University, Guangzhou, China; ^4^Jiangsu Provincial Station of Animal Husbandry, Nanjing, China

**Keywords:** Taihu Lake region, indigenous pig, conservation status, historical relatedness, candidate gene

## Abstract

Chinese indigenous pig breeds in the Taihu Lake (TL) region of Eastern China are well documented by their exceptional prolificacy. There are seven breeds in this region including Meishan (MS), Erhualian (EHL), Jiaxing Black (JXB), Fengjing (FJ), Shawutou (SWT), Mi (MI), and Hongdenglong (HDL). At present, these breeds are facing a great threat of population decline, inbreeding depression and lineage admixture since Western commercial pigs have dominated in Chinese pig industry. To provide better conservation strategies and identify candidate genes under selection for these breeds, we explored genome-wide single nucleotide polymorphism (SNP) markers to uncover genetic variability and relatedness, population structure, historical admixture and genomic signature of selection of 440 pigs representing the most comprehensive lineages of these breeds in TL region in a context of 1228 pigs from 45 Eurasian breeds. We showed that these breeds were more closely related to each other as compared to other Eurasian breeds, defining one of the main ancestral lineages of Chinese indigenous pigs. These breeds can be divided into two subgroups, one including JXB and FJ, and the other comprising of EHL, MI, HDL, MS, and SWT. In addition, HDL was highly inbred whereas EHL and MS had more abundant genetic diversity owing to their multiple conservation populations. Moreover, we identified a list of candidate genes under selection for body size and prolificacy. Our results would benefit the conservation of these valuable breeds and improve our understanding of the genetic mechanisms of body size and fecundity in pigs.

## Introduction

**FIGURE 1:**
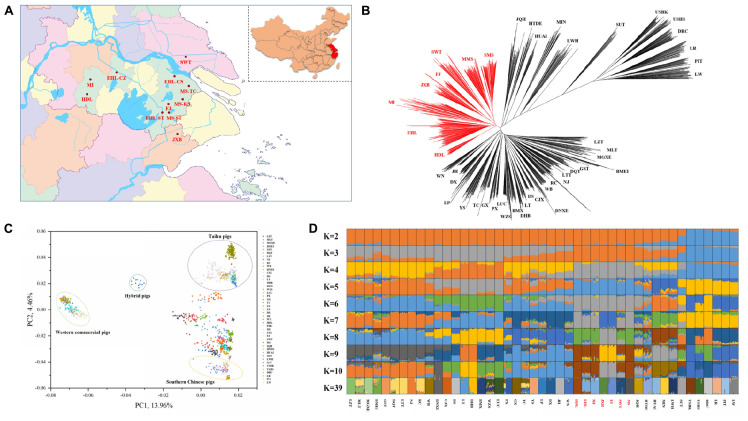
Phylogeny and population structure of 1228 pigs from 45 Eurasian breeds. **(A)** The geographical location of the *in situ* conservation farms for seven indigenous pig breeds in the Taihu Lake region. The seven breeds include Hongdenglong (HDL), Mi (MI), Erhualian (Erhualian in Changzhou, EHL-CZ; Erhualian in Changshu, EHL-CS; Erhualian in Sutai, EHL-ST), Shawutou (SWT), Fengjing (FJ), Jiaxing Black (JXB), and Meishan (Meisha in Taicang, MS-TC; Meishan in Kunshan, MS-KS; and Meishan in Sutai, MS-ST). **(B)** Neighbor-joining tree of 1228 pigs from the 45 breeds. **(C)** Principal component (PC) plots of 1228 pigs from the 45 pig breeds. The first (PC1) and second component (PC2) are shown, the percentage represents the proportion of the corresponding principal component. **(D)** The ancestry of the 45 breeds were analyzed by ADMIXTURE with the assumed number of ancestries (K) from 2 to 10 and 39. Each color represents one ancestral cluster. All breeds are separated by dotted lines. The seven pig breeds in the Taihu Lake region are highlighted in red.

China has a variety of indigenous pig breeds that represent extensive phenotypic diversity and account for approximately one-third of global breeds (Chen et al., 2020). At present, six well-recognized pig breeds are distributed in the Taihu Lake (TL) region in East China (Figure 1A), including Meishan (MS), Erhualian (EHL), Jiaxing Black (JXB), Fengjing (FJ), Shawutou (SWT), and Mi (MI) pigs (China National Commission of Animal Genetic Resources, 2011). Population genomic analyses have shown that the six breeds represent one of the two ancient lineages of Chinese indigenous pigs (Chen et al., 2020). The common features of these six breeds in TL region include resistance to roughage, desirable meat quality and exceptional prolificacy (Haley and Lee, 1993; Hunter et al., 1996; Bosse et al., 2014), which provide valuable genetic resources for further selective breeding of the current commercial breeds (Bosse et al., 2014; Yang et al., 2016). For instance, MS and JXB pigs have been introduced into France to improve reproductive performance of local breeds since the 1980s (China National Commission of Animal Genetic Resources, 2011).

During the past decades, Western commercial pigs have gradually dominated in Chinese pig industry, and the Chinese indigenous pigs including breeds in TL region have deeply decreased in population size (Wang et al., 2018). Consequently, Chinese indigenous pigs are facing a great threat of inbreeding depression (Silió et al., 2013; Saura et al., 2015) due to the reduction in the size of the population. In addition, a few conservation farms may have carried out hybrid experiment with other indigenous breeds or Western commercial pigs to improve pig production performances and meet market demand. Thus, some Chinese indigenous breeds are facing a threat of lineage admixture with other indigenous breeds or Western commercial pigs. To strengthen the protection of indigenous pig breeds, Chinese government has established conservation farms for some indigenous pig breeds. The conservation farm is an *in situ* action to build a genetic reservoir to prevent unwanted lineage admixture with other varieties (Li et al., 2020). To be a national or a provincial conservation farm, it should contain more than 100 sows, no less than 12 boars and no less than 6 subfamilies without consanguinity relationship within three generations. The national or provincial conservation farms should be approved by the National or Provincial Livestock and Poultry Genetic Resources Commission, respectively. To provide better conservation strategies for Chinese indigenous pigs, researchers have explored genome-wide SNP markers to uncover conservation status, genetic relatedness and historical admixture of a number of Chinese pig breeds in various regions including TL region (Ai et al., 2013, 2015; Wang et al., 2018; Xu et al., 2019). However, an in-depth investigation is still required to fully understand the population genetics of Chinese breeds in TL region. For instance, Hongdenglong pig (HDL) is an indigenous breed in TL region (Figure 1A). Nevertheless, it remains a subject of debate whether HDL is a sub-population of Chinese Huai pig indigenous in Anhui and Henan provinces or is an ancestral genetic lineage having close genetic relationship with MI and EHL (Chen et al., 2016). Meishan pigs can be classified into two subpopulations, Small MS (SMS) and Medium MS (MMS), which are characterized by small and large body sizes, respectively; but the genetic mechanism underlying this phenotypic variation remains largely unknown (Sun et al., 2018). So far, more than one conservation farms have been established for MS and EHL in TL region. Nevertheless, the genetic relatedness of MS and EHL individuals in different conservation farms is still elusive. In addition, previous studies (Wang et al., 2015, 2017; Xiao et al., 2017) have examined a limited number of individuals that did not cover all potential consanguinities of each breed in TL region. Since some conservation farms have collected new boars without pedigree records from the origin or distribution areas of these indigenous pig genetic resources to avoid inbreeding depression and these new boars might create new consanguinities. For this reason, this study aimed to unravel genetic variability and relatedness, population structure, historical admixture and genomic selection signature of 440 pigs representing the most comprehensive consanguinities of the seven indigenous breeds in TL region in a context of 45 Eurasian breeds. This work will benefit the efficient conservation and utilization of these breeds in TL region, and our findings will advance our understanding of the genetic mechanisms underlying the germplasm characteristics of these breeds, such as fecundity and body size.

## Materials and Methods

### Animals and SNP Genotyping

A total of 1228 pigs from 45 Eurasian breeds including the seven breeds in TL region were analyzed in this study (Table 1). The seven breeds comprise EHL, MS, FJ, MI, JXB, SWT, and HDL. We collected ear tissue samples of 440 pigs from the seven breeds that were raised in national or provincial conservation farms in the Yangtze river delta (Figure 1A). EHL (*n* = 111) and MS pigs (*n* = 136) were each sampled from three conservation farms while other five breeds were each sampled from one conservation farm. Among the three conservation farms for MS, one is MMS conserved population, the other two are SMS conserved populations. The 440 individuals were selected to cover all consanguinities of the conservation populations of the seven breeds according to their pedigrees. We preferentially selected all boars and unrelated sows without common ancestors within three generations and included all sows of unknown origin. All procedures and animals used in this study were in compliance with guidelines for the care and utility of experimental animals established by the Ministry of Agriculture and Rural Affairs of China. The ethics committee of Nanjing Agricultural University approved this study.

**TABLE 1:** Genetic diversity of 45 Eurasian pig breeds in this study.

Region	Breed	Abbreviation	Number of individuals	Ho	He	Fis	LD (kb)
Taihu Lake region	Hongdenglong	HDL	30	0.16	0.15	0.5	1000
	Erhualian	EHL	111	0.2	0.2	0.4	9.5
	Mi	MI	59	0.23	0.22	0.29	25.5
	Jiaxing Black	JXB	40	0.19	0.19	0.41	261.63
	Fengjing	FJ	23	0.19	0.17	0.41	488.74
	Shawutou	SWT	41	0.26	0.24	0.22	76.54
	Meishan	MS	136	0.18	0.19	0.47	79.54
East China	Wannan spotted	WN	18	0.2	0.2	0.38	190.6
	Jiangquhai	JQH	30	0.24	0.23	0.26	159.58
	Jinhua	JH	13	0.15	0.14	0.54	445.72
	Dongxiang spotted	DX	30	0.18	0.18	0.46	350.68
	Leping spotted	LP	20	0.23	0.22	0.31	46.52
	Yushan Black	YS	36	0.22	0.21	0.33	137.57
Central China	Tongcheng	TC	16	0.22	0.21	0.32	13.51
	Ganxi Two-end-black	GX	13	0.18	0.15	0.46	> 1000
	Pingxiang Two-end-black	PX	35	0.23	0.23	0.29	3.5
South China	Luchuan	LUC	18	0.17	0.16	0.48	53.53
	Wuzhishan	WZS	16	0.25	0.24	0.25	4.5
	Bama Xiang	BMX	16	0.22	0.2	0.34	12.51
	Large Black-white	DHB	16	0.22	0.19	0.33	>1000
	Lantang	LT	20	0.19	0.18	0.43	243.62
	Dongshan	DS	15	0.19	0.17	0.41	122.56
	Congjiang Xiang	CJX	16	0.19	0.17	0.42	397.7
	Diannan Small-ear	DNXE	15	0.19	0.21	0.44	150.58
	Chinese Wild Boar	WB	21	0.22	0.23	0.32	1.5
Southwest China	Rongchang	RC	18	0.2	0.2	0.38	7.5
	Neijiang	NJ	16	0.21	0.2	0.35	98.55
	Litang Tibetan	LTT	16	0.19	0.2	0.55	4.5
	Diqing Tibetan	DQT	19	0.23	0.22	0.3	3.5
	Mingguang Small-ear	MGXE	13	0.28	0.25	0.14	56.53
	Milin Tibetan	MLT	16	0.28	0.27	0.15	7.5
	Linzhi Tibetan	LZT	29	0.24	0.23	0.15	25.51
North China	Bamei	BMEI	16	0.26	0.24	0.22	195.6
	Gansu Tibetan	GST	21	0.22	0.21	0.32	20.51
	Hetao Big-ear	HTDE	16	0.29	0.26	0.1	118.56
	Huai	HUAI	15	0.28	0.22	0.18	61.53
	Min	MIN	22	0.29	0.27	0.12	698.85
	Laiwu	LWH	30	0.28	0.26	0.15	98.55
Hybrid	Sutai	SUT	12	0.33	0.32	−0.02	1000
Western Countries	Berkshire	USBK	20	0.27	0.27	0.18	518.76
	Hampshire	USHS	20	0.31	0.3	0.06	409.7
	Duroc	DRC	35	0.28	0.3	0.14	308.65
	Landrace	LR	35	0.34	0.36	−0.04	185.59
	Pietrain	PIT	20	0.34	0.33	−0.03	500.75
	Large White	LW	35	0.36	0.37	−0.11	182.59

*Ho, observed heterozygosity; He, expected heterozygosity; Fis, inbreeding coefficient; LD, Linkage disequilibrium extent at genotype correlation coefficients *r*^2^ value of 0.3.*

Genomic DNA was extracted from ear samples using a routine phenol/chloroform method and diluted to a final concentration of 50 ng/μl, which was then genotyped using the Porcine SNP50 BeadChip (Illumina, United States), containing 51,315 SNPs according to the manufacturer’s protocol. The genotype input was converted into a PLINK v1.07 (Purcell et al., 2007) input file. SNPs were filtered with single individual call rate of greater than 90%, single SNP call rate of greater than 90% and a minor allele frequency of greater than 0.05. All unmapped SNPs and SNPs on sex chromosomes were discarded. After the filtering process, we obtained a total of 29,754 informative SNPs from the 440 individuals. Subsequently, we retrieved the publicly available 60K SNP data of 788 pigs from other 38 Eurasian breeds (Wang et al., 2018) and merged these datasets into our data under the same quality control conditions. A final set of 18,703 SNPs from 1,228 pigs were used for subsequent statistical analyses. The PLINK format data of these 1,228 samples is available at https://doi.org/10.6084/m9.figshare.10073153.v1.

### Estimation of Population Genetic Differentiation

To estimate the genetic distances between individuals, we calculated the average proportion of alleles shared (Dst) using PLINK v1.07 (Purcell et al., 2007). The genetic distances between all pair-wise individuals were calculated as (1-Dst). A neighbor-joining (NJ) tree was constructed using MEGA v6.0 (Tamura et al., 2013) based on the matrix of (1-Dst) (Ai et al., 2013; Chen et al., 2018) and displayed via FIGTREE v1.4.2^1^. We used MEGA v6.0 (Tamura et al., 2013) to calculate the average distances between pair-wise breeds based on the (1-Dst) matrix.

Coefficients of genetic differentiation (*Fst*) among breeds, also named Wright’s F-derived statistics (Weir and Cockerham, 1984), were calculated using GENEPOP v4.2 (Rousset, 2008). The *Fst* values were identified as follows: 0–0.05 for low, 0.05–0.15 for moderate, 0.15–0.25 for great and above 0.25 for highly genetic differentiations (Li et al., 2014). We constructed heatmaps using the genetic distance (1-Dst) and genetic differentiation coefficient (*Fst*) matrices and visualized these heatmaps via in-house R scripts.

A principal component analysis (PCA) was performed using PLINK v1.07 (Purcell et al., 2007) to illustrate the relationship among breeds. We chose the first two or three principal components to visualize the PCA results via in-house R scripts.

### Inferring of Population Structure and Admixture

ADMIXTURE v1.23 (Alexander et al., 2009) was used to infer the most probable number of ancestral populations (K) based on the SNP genotype data. For the 45 Eurasian breeds, results were plotted for K from 2 to 10 and 39 using in-house R scripts. To obtain comparable results across all breeds and to avoid the potential bias caused by different sample sizes of these breeds, we randomly selected a subset of 12 pigs from each breed for the ADMIXTURE analysis. For the seven breeds in TL region, 23 individuals from each breed were tested and the results were plotted for K from 2 to 7 using in-house R scripts.

TREEMIX v1.12 (Pickrell and Pritchard, 2012) was used to infer historical migration events between pair-wise breeds. European Large White pigs were treated as an outgroup. The block size was set to 200 SNPs to ensure independency between blocks. To verify the consistency of migration edges, 10 independent replicates were run for the model with each migration events.

The three-population test (Reich et al., 2009) implemented in the THREEPOP program of TREEMIX (Pickrell and Pritchard, 2012) was used to detect population admixture. To assess the statistical significance of the three-population test, we used a Block Jackknife test (Kunsch, 1989) with a window of 200 SNPs to obtain the standard error of the test, which was used to generate a *Z* score. A large negative *Z* score (*Z* < -2) indicates a significant evidence of admixture.

### Analysis of Genetic Diversity Indices

Expected heterozygosity (He), observed heterozygosity (Ho), and inbreeding coefficient (Fis) were calculated for individual breed using PLINK v1.07 (Purcell et al., 2007) under default settings. The inbreeding coefficient (Fis) of each breed was calculated based on the observed versus expected number of homozygous genotypes via the following formula (Purcell et al., 2007):

$$F\,\text{is}=\frac{O\,\left(H\,O\,M\right)-E\,\left(H\,O\,M\right)}{N\,\left(N\,M\right)-E\,\left(H\,O\,M\right)}$$

Where Fis is the inbreeding coefficient estimation; O(HOM) is the observed number of homozygotes; E(HOM) is the expected number of homozygotes; N(NM) is the number of non-missing genotypes.

Linkage disequilibrium (LD) extent in each breed was estimated from pair-wise genotype correlation coefficients (*r*^2^) via the command “–r2 –ld-window 9999 –ld-window-r2 0 –ld-window-kb 1000” in PLINK v1.07 (Purcell et al., 2007). Inter-SNP distances (kb) were binned into the following classes: 0–1, 1–3, 3–6, 6–9, 9–15, 15–30, 30–40, 40–60, 60–80, 80–100, 100–150, 150–200, 200–250, 250–300, 300–500, 500–800, and 800–1000 kb. Pair-wise *r*^2^ values were averaged over all 18 autosomes and were plotted as a function of increasing inter-SNP distance. The physical distance at which the pair-wise genotype correlation in the filtered SNP dataset decays was below a threshold of 0.3 (Ai et al., 2013). SNeP (Barbato et al., 2015) was used to estimate historic effective population size (Ne) of each breed using the following formula (Corbin et al., 2012):

$$N_{T\,\left(t\right)}=\frac{1}{\left(4\,f\,\left(C_{t}\right)\right)}\,\left(\frac{1}{E\,\left[r_{a\,d\,j}^{2}|C_{t}\right]}-\alpha \right)$$

Where N_T_(t) is the estimated effective population size t generations ago; c_t_ is the recombination rate t generations ago; *r*^2^_adj_ is the LD estimation adjusted for sampling bias; α is a constant.

### Characterization of Subfamilies of Each Conservation Population

FJ, MI, JXB, SWT, and HDL were each sampled from one conservation farm while three conservation populations were investigated for EHL and MS, respectively. There were historical exchanges of breeding animals among these populations. To clarify the most reliable subfamilies of each conservation farm, we performed neighbor-joining (NJ) tree of all individuals from each farm using MEGA v6.0 (Tamura et al., 2013). The bootstrap was set to 1000 times, and we obtained a fixed NJ tree clustering result. Since we were not sure about the appropriate number of subfamilies, we firstly divided the subfamilies as much as possible based on the clustering pattern of the NJ tree. For individuals adjacent to each other, only the adjacent individuals whose genetic distance were less than two standard deviations compared to the population mean value can be divided into same subfamily. We then calculated the genetic distance (1-Dst) and genetic differentiation (*Fst*) between pair-wise subfamilies and merged adjacent subfamilies with a close genetic distance or low genetic differentiation into a single subfamily, so that the variation coefficient of the genetic distance between pair-wise subfamilies of each population was less than 10%, and the *Fst* values between pair-wise subfamilies of each population were greater than 0.05. In the end, we tried to make the genetic resource of conservation farm evenly distributed between subfamilies, which means the genetic distances between pair-wise subfamilies tended to be consistent, and the genetic differentiation between pair-wise subfamilies should also be moderate degree.

### Detection of Candidate Genes Under Selection

To identify candidate loci for body size differences between MMS and SMS, we calculated genetic differentiation coefficients (*Fst*) (Weir and Cockerham, 1984) of 21,850 informative SNPs which were qualified following the same quality control conditions between 63 MMS individuals and 73 SMS individuals. We ranked these SNPs by their *Fst* values and had a close examination on top 10 SNPs with the highest *Fst* values to identify candidate genes under selection. We searched for candidate genes (within 100-kb regions) and quantitative trait loci (QTL, within 1-Mb regions) flanking these SNPs on the pig reference genome assembly (*Sscrofa* 11.1)^2^ according to the published literatures and gene functional relevance to body size, growth, and development. In addition, we employed PLINK v1.07 (Purcell et al., 2007) to detect correlation coefficients (*r*^2^) between pair-wise top significant SNPs on SSC3.

To identify candidate loci under selection in the seven breeds in TL region, we calculated locus-specific branch length (LSBL) statistics (Shriver et al., 2004) for each polymorphic site of 18,703 qualified SNPs under a three-group contrasting model. According to the clustering pattern in the NJ and TREEMIX trees, we randomly selected 113 individuals from all seven breeds in TL region as Group 1, all 86 pigs from South Chinese breeds (Bamaxiang, Dahuabai, Luchuan, and Wuzhishan pigs) as Group 2, and all 114 individuals from Southwest Chinese breeds (Neijiang, Rongchang and Tibetan pigs) as Group 3. The LSBL statistics were computed as previously reported (Ai et al., 2014). Candidate genes were retrieved within 100-kb regions flanking SNPs with LSBL values at the empirical threshold of top 0.5% distribution. The Gene Ontology (GO) databases were used for the gene functional enrichment analyses of the retrieved genes via David^3^.

## Results

### Phylogenic Relationships and Genetic Differentiation

First, we constructed the NJ tree for all 1228 pigs from 45 Eurasian breeds (Figure 1B). Two HDL pigs and three MI pigs did not cluster with their original breeds and were thus excluded from the subsequent analyses due to their likely admixture. Western commercial pigs and Chinese indigenous pigs formed two major groups and the clustering patterns within each group were consistent with the previous reports (Megens et al., 2008; Ai et al., 2013, 2015; Yu et al., 2013; Yang et al., 2017; Wang et al., 2018). Briefly, pigs in TL region were closely clustered with each other, defined one major clade in Chinese major groups, which showed their genetic differentiation from the other Chinese indigenous pigs. And pig breeds from East China, Central China, South China, and Southwest China were defined another major clade. Within this clade, we can still observe that the indigenous pig breeds in East China, Central China, South China, and Southwest China were more closely clustered with each other respectively. We also observed that JQH clustered closely with pigs in North China, and GST and BMEI clustered with pigs in Southwest China. All HDL pigs clustered together and displayed a close relationship with the other six breeds in TL region but not with Huai pigs, which were assumed to share a common ancestor with HDL pigs (China National Commission of Animal Genetic Resources, 2011).

We also plotted two heatmaps using the genetic distance (1-Dst) and genetic differentiation coefficient (*Fst*) matrices between pair-wise breeds. The two heatmaps (Supplementary Figure S1) consistently showed that the seven breeds in TL region were closely related and had larger genetic distances from other Chinese breeds. Moreover, PC1 separated Western commercial pigs from Chinese indigenous pigs. Also, Sutai pig is a newly cultivated breed, which was bred based on the hybridization of Chinese and Western pigs. Therefore, Sutai pig was located between Western commercial pigs and Chinese indigenous pigs. At the same time, PC2 displayed the genetic differentiation between the seven breeds in TL region and other Chinese breeds in the PCA plots (Figure 1C). This is consistent with the patterns in the NJ tree and heatmaps.

**FIGURE 2:**
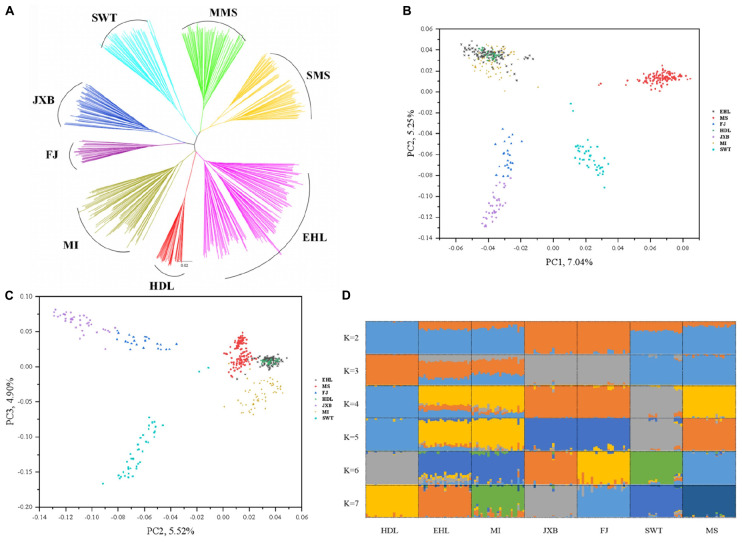
Phylogeny and population structure of seven pig breeds in the Taihu Lake region. **(A)** Neighbor-joining tree of the seven breeds. **(B)** Principal component (PC) plots of the seven pig breeds. The first (PC1) and second component (PC2) are shown, the percentage represents the proportion of the corresponding principal component. **(C)** Principal component (PC) plots of the seven breeds. The second component (PC2) and third component (PC3) are shown, the percentage represents the proportion of the corresponding principal component. **(D)** The ancestral compositions of the seven breeds were analyzed using ADMIXTURE with the assumed number of ancestries (K) from 2 to 7. Each color represents one ancestral cluster.

To assess the evolutionary origin of these seven breeds in TL region in a context of diverse populations, we conducted the ADMIXTURE analysis for the 45 Eurasian breeds with *K* values ranging from 2 to 10 and 39 (Figure 1D). As previously reported (Ai et al., 2013; Yang et al., 2017; Wang et al., 2018), Chinese and Western pig breeds showed distinct ancestral lineages at *K* = 2. The seven breeds in TL region stood out as one of two ancestral genetic components of Chinese indigenous pigs at *K* = 3. At *K* = 7, EHL, MI, HDL, and MS had major genetic components of the same ancestral lineage while FJ and JXB represented another ancestral lineage, and SWT appeared as a mixture of these two lineages. The lowest cross-validation error was observed at *K* = 39 (Supplementary Figure S2). At this *K* value, JXB, FJ, SWT, MS, MI, and HDL defined different lineages while EHL had a mixed genetic component of HDL and MI (Figure 1D). When we focused on the seven breeds in TL region only, the neighbor-joining (Figure 2A), PCA (Figures 2B,C) and ADMIXTURE (Figure 2D) analyses also indicated that HDL, MI, and EHL were more closely related while FJ and JXB appeared as another subgroup. This was also supported by the genetic distance (1-Dst) and *Fst* matrices among these seven breeds (Supplementary Tables S1, S2). It can be seen that HDL can define a differentiated cluster and different ancestral lineages through multiple analysis methods (Figures 1B,D, 2A,D). We also found that the SMS and EHL were grouped into two and three clusters, respectively (Figure 2A). By verifying the individuals ID, we confirmed that the individuals in these two clusters of SMS were from the two conservation farms including MS-TC and MS-ST, and the individuals in the three clusters of EHL were from the three conservation farms including EHL-ST, EHL-CS, and EHL-CZ. It showed that there were still some differences between different conservation populations within the same pig breed.

**FIGURE 3:**
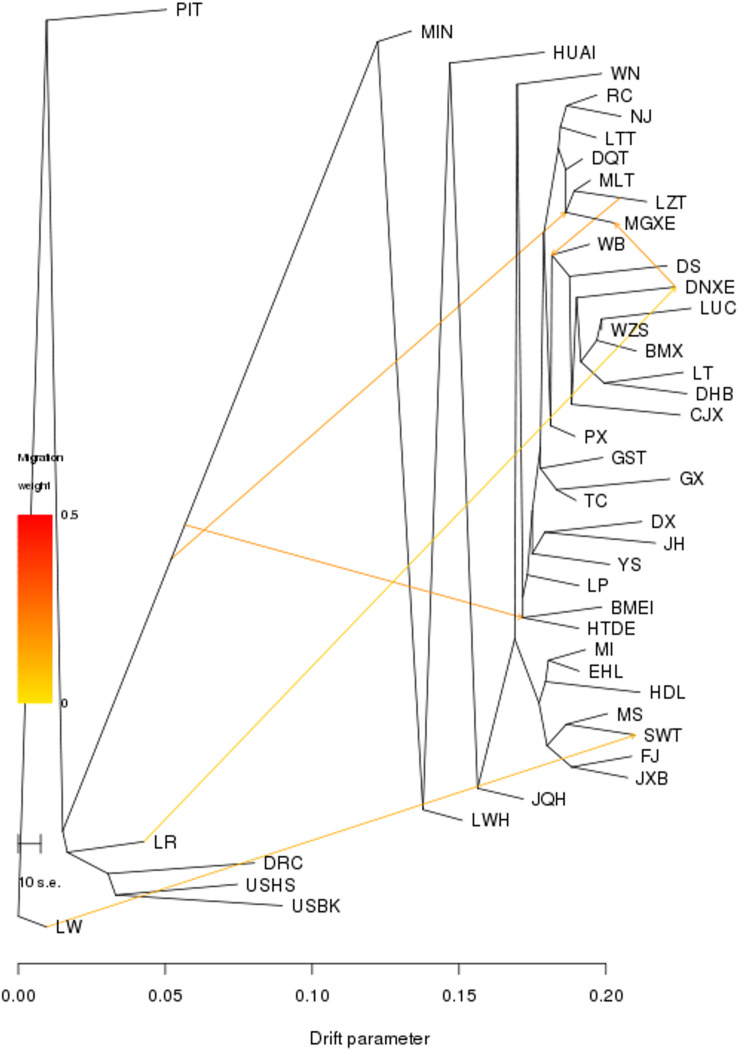
Population splits and admixture of all 45 Eurasian pig breeds were inferred using TREEMIX. Arrows indicate migration events. A spectrum of heat colors indicates different migration weights in the migration event. Horizontal branch lengths are proportional to the amount of genetic drift that occurred on the branch. The scale bar shows 10 times the average standard error of the entries in the sample covariance matrix.

We then conducted the TREEMIX analysis to unravel migration events occurring among all 45 breeds. We set the migration events as 6, at which nearly all (99.98%) of the variance in the relatedness between breeds were explained (Figure 3). Of the seven breeds in TL region, only SWT showed a signature of introgression from European Large White (migration weight = 34.9%). We further conducted a three-population test and found that no hybridization event occurred between these seven breeds in TL region and exotic breeds, except that few SWT individuals showed admixture with exotic lineages (Supplementary Table S3).

### Genetic Diversity Indices

**FIGURE 4:**
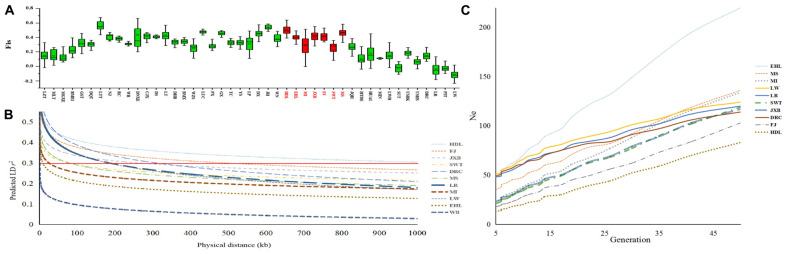
Genetic diversity of seven pig breeds in the Taihu Lake region. **(A)** Each histogram denotes the inbreeding coefficient (Fis) of each breed. Pig breeds are plotted along the *x*-axis while Fis values are plotted along the *y*-axis. Seven pig breeds in the Taihu Lake region are highlighted in red. **(B)** Linkage disequilibrium (LD) extents, that were plotted as a function of inter-SNP distance for the seven pig breeds in the Taihu Lake region, Chinese wild boars (WB) and three representative Western breeds including Landrace (LR), Large White (LW), and Duroc (DRC). Physical distances are plotted along the *x*-axis while LD extents, which were predicted by *r*^2^ were plotted along the *y*-axis. The red solid line indicates the threshold of 0.3. **(C)** Effective population sizes (Ne) of the seven pig breeds in the Taihu Lake region and three representative Western breeds including Landrace (LR), Large White (LW) and Duroc (DRC). Generations are plotted along the *x*-axis while Ne are plotted along the *y*-axis.

To compare genetic variability of the seven breeds in TL region, we calculated three genetic diversity indices for these breeds: Ho, He and Fis. Of note, HDL had the lowest Ho (0.16) and He (0.15) values but a highest Fis estimation (0.50). In contrast, EHL had the larger Ho (0.2) and He (0.2) values but a lower Fis level (0.4). We noticed that several MI pigs were highly inbred, leading to a large coefficient (44.15%) of variation with a relatively small average Fis value of 0.29 (Figure 4A).

We estimated LD extents at the threshold of *r*^2^_0.3_ in all 45 breeds. We chose Chinese wild boars, the seven breeds in TL region and three Western commercial breeds to visualize their LD patterns (Figure 4B). Chinese wild boars (WB) showed the shortest extent of LD (*r*^2^_0.3_ = 1.5 kb). Among these seven breeds in TL region, HDL had the longest LD extents (*r*^2^_0.3_ = 1000.00 kb), followed by FJ (488.74 kb) and JXB (261.63 kb). In comparison, EHL (9.50 kb) and MI (25.50 kb) had the shortest LD extents.

We estimated historic effective population size (Ne) of each breed dating back 50 generations ago using SNeP (Barbato et al., 2015; Figure 4C). EHL had the largest Ne values among these seven breeds in TL region, and showed larger Ne values than Western commercial pigs from 50 till 6 generations (50 years) ago. However, the effective population sizes of all seven breeds in TL region declined dramatically during the past decades, which were even smaller than those of Western commercial pigs at present.

### Population Structure and Subfamilies

Three MS populations were investigated in this study, including one MMS (MS in Kunshan, MS-KS) and two SMS (MS in Sutai, MS-ST and MS in Taicang, MS-TC) populations. Individuals from the three populations did not cluster together and defined separate branches in the NJ tree (Supplementary Figure S3). According to the clustering patterns in the NJ tree, the numbers of subfamilies in MS-KS, MS-ST, and MS-TC were 10, 6, and 6, respectively (Supplementary Figure S4 and Supplementary Table S4). Among these 22 subfamilies, 17 subfamilies contain boars according to the classification method (see section “Materials and Methods”).

We also analyzed three EHL populations that were raised in Changzhou (EHL-CZ), Changsu (EHL-CS) and Sutai (EHL-ST) cities of Jiangsu province. Individuals from each EHL population did not cluster together in the NJ tree (Supplementary Figure S3). A few number of EHL-CZ and EHL-CS individuals were grouped together, which is likely due to the occasional exchange of semen of breeding boars between the EHL-CZ and EHL-CS populations. Nearly all EHL-ST individuals defined a separate branch, suggesting that limit gene flow between EHL-ST and the other two EHL populations. According to the classification method (see section “Materials and Methods”), the number of subfamilies was 8 in each these three populations (Supplementary Figure S4 and Supplementary Table S4) and 18 out of these 24 subfamilies have boars. And the numbers of subfamilies in SWT, MI, FJ, JXB and HDL were 8, 7, 6, 9, and 6, respectively (Supplementary Figure S4 and Supplementary Table S4). The *Fst* values between pair-wise subfamilies of the seven breeds in TL region were all greater than 0.05 (data not shown), indicating a moderate genetic differentiation at the subfamily level.

### Candidate Genes Under Selection for Body Size in Meishan Pigs

**FIGURE 5:**
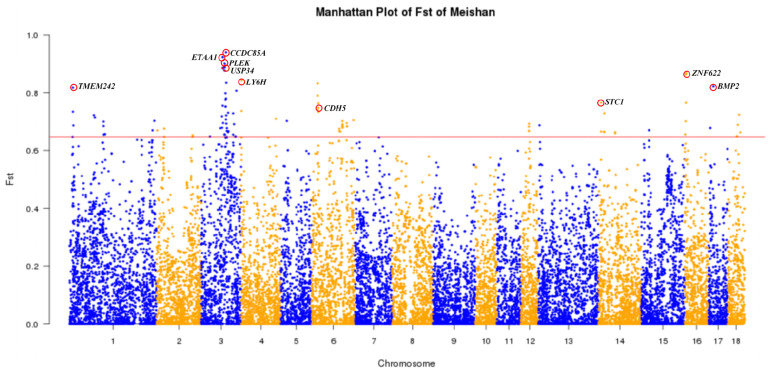
Genome-wide distribution of *Fst* values of 21,850 SNPs between Medium Meishan pigs and Small Meishan pigs. The 18 autosomes are plotted along the *x*-axis while genetic differentiation coefficient (*Fst*) values are plotted along the *y*-axis. Chromosomes are indicated by different colors and the threshold (top 0.5%) indicating signature of selection is denoted with a red solid line. Top ten candidate genes are marked with red circles.

MMS and SMS are two closely related populations of the Meishan breed. Here we used a *Fst*-based approach (see section “Materials and Methods”) to identify candidate genes for the phenotypic difference in body size between these two populations. We identified 10 candidate genes (Figure 5 and Supplementary Table S5), including three brain and nerve development related genes (*CCDC85A*, *LY6H*, and *TMEM424*), four genes involved in bone formation (*USP34*, *BMP2*, *CDH5*, and *STC1*) and three genes associated with disease resistance (*ETAA1, PLEK*, and *ZNF622*). Interestingly, *USP34* is required for osteogenic differentiation and bone formation (Guo et al., 2018), *BMP2* encodes bone morphogenetic protein 2 and regulates osteoinduction both *in vitro* and *in vivo* (Wang D. et al., 2019; Teng et al., 2019), *CDH5* in acetabular labrum cells may be involved in the pathogenesis of osteoarthritis (Wang S. et al., 2019), and up-regulation of *STC1* expression is associated with the occurrence of osteoarthritis in humans (Wu et al., 2019). Moreover, the most prominent region of genetic differentiation between MMS and SMS was located around 84,628,478 bp on SSC3, which is proximal to the *USP34* gene. Importantly, *USP34* modulates osteogenic differentiation and bone formation by regulating *BMP2* signaling gradients (Guo et al., 2018). We further conducted the LD analysis for a 1 Mb region flanking the top SNPs on SSC3, and found that nine SNPs within this region were in nearly complete LD (Supplementary Table S6). Moreover, MMS and SMS were fixed for either “GGAGGACAA” or “AAGAAGATG” haplotype. This supports that haplotypes of this region have experienced directional selection in MS. Moreover, we found that this region resided in multiple QTLs for body weight, obesity index, feed intake and feed conversion ratio (Supplementary Table S7) that have been deposited in the pig QTL database^4^.

### Candidate Genes Under Selection in Chinese Pigs in Taihu Lake Region

**FIGURE 6:**
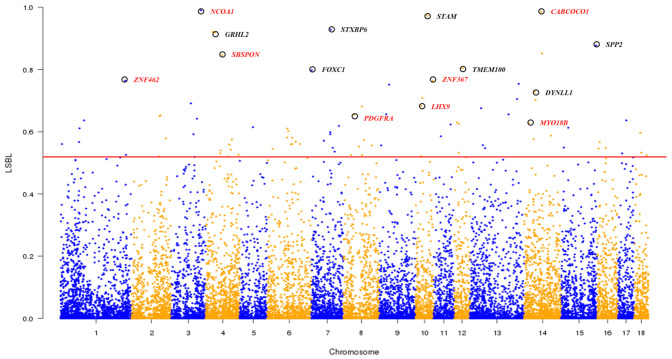
Genome-wide distribution of LSBL values across all 18 autosomes. The 18 autosomes are plotted along the *x*-axis while locus-specific branch length (LSBL) values are plotted along the *y*-axis. Chromosomes are indicated by different colors, and the threshold (top 0.5%) indicating signature of selection in seven indigenous pig breeds in the Taihu Lake region is denoted with a red solid line. Top 15 candidate genes are marked with black circles and eight reproduction-related candidate genes are indicated by red colored words.

As shown in the Admixture analysis at *K* = 4 (Figure 1D), South Chinese, Southwest Chinese and East Chinese (mainly in TL region) breeds are three representative lineages of Chinese indigenous pigs. We conducted the LSBL analysis (see section “Materials and Methods”) to identify genomic loci, at which the seven breeds in TL region showed significant genetic differentiation from both Southwest Chinese breeds and South Chinese breeds. These loci could play a role in the formation of germplasm characteristics of the seven breeds in TL region, such as fecundity. We considered 91 SNPs with the highest (top 0.5%) LSBL values as potential loci under selection and 41 protein-encoding genes within 100-kb regions flanking the 91 SNPs as candidate genes under selection in these seven breeds in TL region (see section “Materials and Methods”). Of note, eight genes at the top 15 loci (Figure 6 and Supplementary Table S8) have been implicated in the reproductive process, including *NCOA1*, *CABCOCO1*, *SBSPON*, *ZNF462*, *ZNF367*, *LHX9*, *PDGFRA*, and *MYO18B*. For instance, *NCOA1* regulates placental morphogenesis and embryo survival (Chen et al., 2010). *CABCOCO1* modulates sperm flagellar movement (Kawashima et al., 2016). *SBSPON* plays a role in the pathogenesis of pre-eclampsia, and could be acted as a biomarkers of pregnancy complication (Kaartokallio et al., 2015). *ZNF462* is essential for embryonic development (Weiss et al., 2017). *ZNF367* is involved in the proliferation of sperm cell (Fu et al., 2018). *LHX9* is indispensable for testis development and testosterone production (Hu et al., 2018). *PDGF* receptor mediates gap junctions in corpus cavernosum smooth muscle cells to regulate male erectile function (Zhang et al., 2018). The variations of *MYO18B*, including both epigenetic and genetic alterations, play an important role in ovarian carcinogenesis (Yanaihara et al., 2004). Moreover, the 41 candidate genes under selection were enriched in five GO biological processes (Supplementary Table S9), including cell proliferation (GO:0008283), phosphatidylinositol-mediated signaling (GO:0048015), Notch signaling pathway (GO:0007219), regulation of smooth muscle contraction (GO:0006940) and ATP binding (GO:0005524). These biological processes, especially cell proliferation, are known to be significantly up-regulated in human ovarian granulosa cells (Kranc et al., 2019).

## Discussion

### Historical Relatedness and Admixture

In this study, we conducted genetic relationships (neighbor-joining tree), PCA, ADMIXTURE, TREEMIX and three-population test analyses to reveal historical relatedness and admixture of seven breeds in TL region and other 38 diverse Eurasian pig breeds. It could be found that, in the NJ trees of 45 Eurasian pig breeds, pig breeds were clustered with each other owing to the same geographic origin, expect JQH, GST and BMEI. The possible reasons for this result are as follows: (1) JQH is located on the border of East China and North China, and it has a certain lineage from HUAI pigs (China National Commission of Animal Genetic Resources, 2011); (2) Both GST and BMEI are located in Northwest China, while the remaining northern pig breeds are concentrated in Northeast China; (3) The habitats of GST and BMEI are closer to the pig breeds in Southwest China, and GST is also a population of Tibetan pigs, clustering with other Tibetan pigs is in line with expectations. We also showed that these seven breeds are more closely related to each other as compared to other Eurasia breeds, as they were grouped together in the PCA plots and defined a separate clade in the neighbor-joining tree. They also had a common ancestral lineage at *K* = 3–6 in the ADMIXTURE analysis. In TREEMIX analysis, for the pig breeds in TL region, there was only one migration event from LW to SWT, and the migration weight was not high, only 34.9%. Therefore, in order to determine whether SWT was mixed with external lineage, we conducted three-population test and found that there was no lineage admixture event related to SWT, whose *Z*-score were all greater than −2 (data not shown) (Kunsch, 1989). Archeological evidence (Qian et al., 2010) suggested that pigs were domesticated in TL region approximately 7,000 years ago. The Liangzhu Culture and Hemudu Culture had been dominating in this region during the period of 5000–7000 years ago. The remains of ancient domestic pigs and ceramic pendants printed with pig patterns have been unearthed in relics of these two cultures (Wang, 1981). Our findings and the previous archeological data seem to support that TL region was one of the domestication sites of Chinese pigs. The supporting data are: (1) In the NJ tree, these seven pig breeds in TL region were clustered into relatively differentiated cluster; (2) In the PCA analysis, the principal component PC2 can clearly distinguish these seven pig breeds in TL region from other Chinese indigenous pig breeds; (3) In the ADMIXTURE analysis of 45 Eurasian pig breeds, these seven pig breeds showed a completely different composition of ancestry lineage from all other pig breeds, indicating they would experience a unique evolutionary route; (4) TREEMIX analysis and three-population test analysis showed that these seven pig breeds did not have lineage introgression from other Eurasian pig breeds, which indicates the purity of their lineage; (5) There have been relevant archeological evidences can prove that Chinese Taihu Lake region has a history of domesticating pigs as early as 7000 years ago, which is one of the earliest areas for domesticating pigs in China. However, further investigations of ancient DNA are required to validate this claim.

Our population genetics data indicated that these seven breeds in TL region can be divided into two subgroups. One includes JXB and FJ, and the other comprises EHL, MI, HDL, MS, and SWT. JXB and FJ have the closest genetic relationship among these seven breeds, and define an independent ancestral lineage at *K* = 7 in the ADMIXTURE analysis, while South Chinese and Southwest Chinese breeds can only set one ancestral lineage at *K* = 7, and these two subgroups can be observed until *K* = 18. These two breeds were originally distributed in a neighboring area (Figure 1A). Among the other five breeds, EHL and MI showed the closest genetic relationship, which is shown in the PCA plots, NJ tree and two heatmaps. This is consistent with the history that EHL was derived from a backcross between MI and the extinct Dahualian pigs (China National Commission of Animal Genetic Resources, 2011). And in Figure 2A, we found SMS and EHL were grouped into two and three clusters, respectively. This result is mainly due to their different conservation farms. Given that conservation farms adopt the *in situ* conservation model, the individuals differentiation among different conservation farms is likely due to their relatively distinct geographical locations. Moreover, the analysis of genetic relatedness of MS and EHL individuals in different conservation farms (Supplementary Figure S3) also showed this sub-structural trend, and it indeed proves that there should be a certain differentiation between the conserved populations of the same breed and this would be a positive signal for conservation procedure.

It remains a long-term debate whether HDL is a sub-population of Huai pigs indigenous in Anhui and Henan provinces or is an independent genetic resource having close genetic relationship with MI and EHL (Chen et al., 2016). Here we present a comprehensive data of population genetics, supporting that the HDL is an independent breed of the geographic group encompassing other six well-recognized breeds in TL region. The supporting data include that (1) all HDL individuals clustered together and formed a branch in the major clade comprising the other six TL region breeds in the NJ and TREEMIX trees; (2) HDL had smaller genetic distances (1-Dst) and less genetic differentiation (*Fst*) with other six breeds in TL region (especially MI and EHL), in comparison with the other Chinese breeds including Huai pigs; and (3) HDL displayed a common ancestral lineage with other six breeds in TL region in the ADMIXTURE analysis when *K* = 3–6 in the panel of the 45 Eurasian breeds.

### Conservation Status and Sustainable Strategy

We calculated inbreeding indicators, effective population sizes and LD extents of the seven breeds in TL region. We also divided subfamilies within each conservation population. These analyses can reflect the genetic diversity of breed. In fact, different subfamilies of conservation population represent different lineages. The different subfamilies evidenced among the breeds are mainly due to the differences in breeding plans in each conservation farm. Specifically, the biggest effect of dividing subfamilies is that it can be efficient to carry out rotational crossbreeding (Hale and Bondari, 1986; Kuhlers et al., 1994) between different subfamilies, which can prevent breeding within subfamily and reduce inbreeding. Our results indicated that HDL was highly inbred, as this breed had the smallest He, Ho and Ne values but the largest Fis value and the longest LD extents among the seven breeds, it also had the fewest number of subfamilies. All indicators showed that HDL had the lowest genetic diversity. Also, after statistical comparison, the Ho, He, and Fis values of HDL were significantly lower than those of other pig breeds in TL region (ANOVA, *P* < 0.01), such as MS, FJ, and JXB. To avoid deteriorative inbreeding depression, particular attention should be paid to improve the management of the conservation population of this breed. In comparison, EHL had more abundant genetic diversity, because it had lower inbreeding degree, largest effective population size, lowest linkage disequilibrium and the largest number of subfamilies. So did MS, the degree of inbreeding may be relatively high among seven pig breeds in TL region, but it had lower linkage disequilibrium, and it also had the second largest effective population size and subfamilies number. These factors implied that EHL and MS, especially EHL, had more genetic diversity, which is likely due to the multiple conservation farms established for these two breeds. Currently, African swine fever has swept across China and caused disastrous effects on Chinese pig industry. To deal with the devastating disease, it is highly necessary to provide multiple conservation sites for HDL, MI, SWT, JXB, and FJ as well as other Chinese breeds. There would be otherwise a great risk of total destruction for these breeds when the current single *in situ* conservation farm is infected with African swine fever.

### Candidate Genes for the Breed Characteristics

We first performed the *Fst* analysis between MMS and SMS and identified four candidate genes related to bone formation, including *USP34*, *BMP2*, *CDH5*, and *STC1*. And we also found multiple QTLs for body weight, obesity index, feed intake and feed conversion ratio near the most significant locus on SSC3. These candidate genes and QTL intervals may explain the genetic mechanisms underlying the phenotypic difference in body size between MMS and SMS. Sun et al. (2018) also reported several candidate genes for body size phenotypic variation using a genotyping-by-sequencing approach for MMS and SMS. Some of these genes such as *PACSIN1* and *SPDEF* were not replicated in this study. This inconsistency may be due to different experimental populations or different SNP systems. In additional, Sun et al. (2018) detected a significant locus for this phenotypic variation with a top SNP at 66,953,525 bp on SSC3, while we observed the most prominent signal at a different position (84,628,478), which is proximal to the *USP34* gene for bone formation (Guo et al., 2018) on the same chromosome.

The indigenous pig breeds in TL region are well known for their high fecundity with an average litter size of greater than 15 (He et al., 2017). We implemented the LSBL approach to identify a list of candidate genes for the fecundity of TL region breeds. These genes play a role in embryonic development and survival (*NCOA1*, *ZNF462*), pregnancy (*SBSPON*), spermatogenesis and male function (*CABCOCO1*, *ZNF367*, *LHX9*, *PDGFRA*) and ovarian lesions (*MYO18B*). Moreover, these candidate genes are enriched in several GO terms associated with reproduction. These terms include cell proliferation, which is critical for the formation of sperm and eggs (Kranc et al., 2019), phosphatidylinositol-mediated signaling which is involved in mammalian ovarian follicular development (Wu et al., 2013), notch signaling pathway which regulates the ovine follicular development (Jing et al., 2017) and ATP binding which is important for sperm flagellum dynamics (Inoue and Shingyoji, 2007). These findings advance our understanding of the molecular mechanisms underlying the reproductive performance in pigs.

## Conclusion

Our study uncovered genetic diversity, historical relatedness and population structure of seven pig breeds in TL region via a more comprehensive manner. We showed that HDL is an independent genetic resource and the seven breeds in TL region including HDL comprise one of the major ancestral lineages of Chinese indigenous pigs. We further identified a list of novel candidate genes contributing to the phenotypic difference in body size between SMS and MMS, and the fecundity of the seven breeds in TL region. Altogether, our results would benefit the sustainable conservation of these pig breeds in TL region and improve our understanding of the genetic mechanisms of body size and fecundity in pigs.

## Data Availability Statement

We have submitted our SNP dataset to a public repository, which is now publicly available at https://doi.org/10.6084/m9.figshare.10073153.v1.

## Ethics Statement

The animal study was reviewed and approved by the Ethics Committee of Nanjing Agricultural University.

## Author Contributions

RH, JR, and PL: conceptualization. CL and PL: formal analysis. CL, PL, WZ, XM, XW, YX, NJ, MZ, TZ, and YY: investigation. JR, RH, CL, and PL: methodology. RH and JR: project administration. CL and PL: writing – original draft. JR and RH: writing – review and editing.

## Conflict of Interest

The authors declare that the research was conducted in the absence of any commercial or financial relationships that could be construed as a potential conflict of interest.

## Footnotes

1 http://tree.bio.ed.ac.uk/software/figtree

2 www.ensembl.org

3 https://david.ncifcrf.gov

4 https://www.animalgenome.org/cgi-bin/QTLdb/SS/index
