# Supplementary material for: Genome Data Uncover Conservation Status, Historical Relatedness and Candidate Genes Under Selection in Chinese Indigenous Pigs in the Taihu Lake Region
Source: Front Genet. 2020 Jun 9;11:591. doi: 10.3389/fgene.2020.00591 (PMC7296076; doi:10.3389/fgene.2020.00591)
Supplement: Supplementary file 1 [file Presentation_1.pdf]

Table S1. Genetic distances (1-Dst) between pair-wise breeds of seven indigenous pig breeds in the Taihu Lake region.

	EHL	HDL	MI	FJ	JXB	SWT	MS
EHL							
HDL	0.282						
MI	0.282	0.299					
FJ	0.293	0.305	0.311				
JXB	0.304	0.309	0.317	0.278			
SWT	0.320	0.327	0.330	0.323	0.326		
MS	0.294	0.302	0.307	0.307	0.314	0.302	

The full name of each breed is given in Table 1.

Table S2. Genetic differentiation coefficient (*Fst*) between pair-wise breeds of seven indigenous pig breeds in the Taihu Lake region.

	EHL	HDL	MI	FJ	JXB	SWT	MS
EHL							
HDL	0.174						
MI	0.100	0.207					
FJ	0.173	0.298	0.206				
JXB	0.190	0.291	0.214	0.187			
SWT	0.163	0.247	0.173	0.207	0.211		
MS	0.132	0.223	0.151	0.205	0.212	0.146	

The full name of each breed is given in Table 1.

Table S3. Three-population test for Chinese and Western pig breeds.

Target populations	Admixed populations	f3 statistic	Z-score
HTDE	DRC, EHL	-0.014	-12.076
HTDE	DRC, GX	-0.015	-12.022
HTDE	DRC, HDL	-0.013	-11.837
HTDE	DRC, JH	-0.013	-11.606
HTDE	DRC, MS	-0.013	-10.890
HTDE	DS, USBK	-0.013	-10.868
HTDE	GX, USBK	-0.012	-10.524
HTDE	GX, USHS	-0.014	-10.397
HTDE	JH, LR	-0.011	-9.991
HTDE	JH, LW	-0.012	-9.662
HTDE	JH, PIT	-0.013	-9.256
HTDE	JH, USBK	-0.012	-8.659
HTDE	JH, USHS	-0.012	-8.100
HTDE	LR, EHL	-0.009	-7.964
HTDE	LR, HDL	-0.008	-7.729
HTDE	LTT, USBK	-0.010	-7.707
HTDE	LW, EHL	-0.007	-7.284
HTDE	LW, HDL	-0.010	-7.226
HTDE	PIT, EHL	-0.010	-7.186
HTDE	PIT, HDL	-0.011	-6.981
HTDE	PIT, MS	-0.011	-6.868
HTDE	RC, USBK	-0.006	-6.800
HTDE	RC, USHS	-0.009	-6.774
HTDE	TC, USBK	-0.009	-6.747
HTDE	USBK, DX	-0.007	-6.731
HTDE	USBK, EHL	-0.008	-6.702
HTDE	USBK, HDL	-0.007	-6.674
HTDE	USBK, MS	-0.009	-6.485
HTDE	USHS, DX	-0.006	-6.417
HTDE	USHS, EHL	-0.008	-6.330
HTDE	USHS, HDL	-0.009	-6.291
HTDE	USHS, MS	-0.007	-6.251
MGXE	DRC, USBK	-0.008	-6.202
MLT	BMX, USBK	-0.011	-6.192
MLT	BMX, USHS	-0.010	-6.123
MLT	CJX, DRC	-0.008	-5.922
MLT	CJX, LR	-0.009	-5.914
MLT	CJX, LW	-0.011	-5.864
MLT	CJX, PIT	-0.008	-5.856
MLT	JX, USBK	-0.008	-5.762
MLT	JX, USHS	-0.009	-5.741
MLT	DQT, DRC	-0.008	-5.704

MLT	DQT, LR	-0.010	-5.563
MLT	DQT, LW	-0.009	-5.557
MLT	DQT, PIT	-0.009	-5.463
MLT	DQT, USBK	-0.008	-5.070
MLT	DQT, USHS	-0.007	-5.039
MLT	DRC, DS	-0.007	-5.033
MLT	DRC, DX	-0.008	-4.837
MLT	DRC, EHL	-0.007	-4.810
MLT	DRC, GST	-0.008	-4.801
MLT	DRC, GX	-0.007	-4.796
MLT	DRC, HDL	-0.008	-4.637
MLT	DRC, JH	-0.007	-4.537
MLT	DRC, LT	-0.007	-4.530
MLT	DRC, LTT	-0.008	-4.501
MLT	DRC, LUC	-0.008	-4.421
MLT	DRC, PX	-0.007	-4.407
MLT	DRC, RC	-0.007	-4.327
MLT	DRC, TC	-0.007	-4.286
MLT	DS, LR	-0.008	-4.149
MLT	DS, LW	-0.006	-4.051
MLT	DS, PIT	-0.007	-4.032
MLT	DS, USBK	-0.006	-3.991
MLT	DS, USHS	-0.005	-3.933
MLT	GST, USBK	-0.006	-3.927
MLT	GST, USHS	-0.007	-3.860
MLT	GX, LR	-0.007	-3.802
MLT	GX, LW	-0.005	-3.798
MLT	GX, PIT	-0.007	-3.794
MLT	GX, USBK	-0.007	-3.776
MLT	GX, USHS	-0.007	-3.718
MLT	JH, LR	-0.007	-3.643
MLT	JH, LW	-0.006	-3.629
MLT	JH, PIT	-0.008	-3.624
MLT	JH, USBK	-0.007	-3.615
MLT	JH, USHS	-0.005	-3.601
MLT	LR, DX	-0.006	-3.589
MLT	LR, EHL	-0.009	-3.577
MLT	LR, HDL	-0.006	-3.558
MLT	LR, LTT	-0.006	-3.498
MLT	LR, LUC	-0.006	-3.485
MLT	LR, NJ	-0.006	-3.431
MLT	LR, RC	-0.006	-3.332
MLT	LR, TC	-0.006	-3.326
MLT	LTT, LW	-0.005	-3.225
MLT	LTT, PIT	-0.006	-3.217

MLT	LTT, USBK	-0.004	-3.117
MLT	LTT, USHS	-0.005	-3.079
MLT	LUC, LW	-0.006	-3.061
MLT	LUC, PIT	-0.007	-3.018
MLT	LUC, USBK	-0.006	-2.986
MLT	LUC, USHS	-0.004	-2.937
MLT	LW, DX	-0.005	-2.879
MLT	LW, EHL	-0.005	-2.863
MLT	LW, GST	-0.005	-2.852
MLT	LW, HDL	-0.004	-2.802
MLT	LW, LT	-0.005	-2.765
MLT	LW, NJ	-0.006	-2.733
MLT	LW, PX	-0.005	-2.701
MLT	LW, RC	-0.004	-2.693
MLT	LW, TC	-0.004	-2.676
MLT	NJ, PIT	-0.004	-2.656
MLT	NJ, USBK	-0.006	-2.634
MLT	NJ, USHS	-0.004	-2.564
MLT	PIT, DX	-0.005	-2.533
MLT	PIT, EHL	-0.005	-2.527
MLT	PIT, HDL	-0.005	-2.510
MLT	PX, PIT	-0.005	-2.487
MLT	PX, USBK	-0.005	-2.467
MLT	PX, USHS	-0.004	-2.462
MLT	RC, PIT	-0.005	-2.427
MLT	RC, USBK	-0.005	-2.375
MLT	RC, USHS	-0.005	-2.368
MLT	TC, PIT	-0.004	-2.367
MLT	TC, USBK	-0.004	-2.350
MLT	TC, USHS	-0.004	-2.348
MLT	USBK, DX	-0.003	-2.295
MLT	USBK, EHL	-0.004	-2.246
MLT	USBK, HDL	-0.004	-2.240
MLT	USBK, LT	-0.004	-2.219
MLT	USHS, DX	-0.003	-2.184
MLT	USHS, EHL	-0.003	-2.169
MLT	USHS, HDL	-0.004	-2.155
MLT	USHS, LT	-0.004	-2.108
WZS	DRC, LUC	-0.003	-2.100
WZS	LR, LUC	-0.004	-2.067
WZS	LUC, LW	-0.004	-2.033
WZS	LUC, PIT	-0.003	-2.019
WZS	LUC, USBK	-0.005	-2.013
WZS	LUC, USHS	-0.003	-2.006

The presented are all populations with at least one significantly negative f3 statistic

(Z-score < -2). The populations labelled as “Target populations” are the putative mixing population. The populations labelled as “Admixed populations” are those that give the most significantly negative f3 statistic, and are not necessarily the historically admixed populations.

Table S4. The number of subfamilies in each conservation farm.

Conservation farm	Genetic distance between pair-wise subfamilies		Number of subfamilies	
	Mean ± SD	Variable coefficient (%)	Number of boar subfamilies	Number of total subfamilies
HDL	0.16 ± 0.01	8.03	4	6
MI	0.23 ± 0.01	4.51	6	7
FJ	0.18 ± 0.01	7.13	5	6
JXB	0.19 ± 0.01	6.49	7	9
SWT	0.24 ± 0.02	9.99	5	8
EHL-ST	0.20 ± 0.01	5.48	6	8
EHL-CS	0.22 ± 0.01	4.73	6	8
EHL-CZ	0.23 ± 0.01	3.24	7	8
MS-KS	0.19 ± 0.01	5.51	8	10
MS-TC	0.18 ± 0.01	4.36	4	6
MS-ST	0.17 ± 0.02	12.23	5	6

The full name of each breed is given in Table 1.

Table S5. Details of the most significant 10 SNPs with the highest *Fst* values.

Chr	Position (bp)	ID	Location	Closest gene	*Fst* value
3	84628478	rs341843841	Intergenic variant	*CCDC85A*	0.940
3	74636717	rs81372698	Intergenic variant	*ETAA1*	0.924
3	73915766	rs81286186	Intronic variant	*PLEK*	0.923
3	80405782	rs342302874	Intronic variant	*USP34*	0.900
16	5632753	rs81328211	Intergenic variant	*ZNF622*	0.868
4	1232672	rs81325155	Intergenic variant	*LY6H*	0.842
17	15949323	rs80919610	Intergenic variant	*BMP2*	0.822
1	9755372	rs80805707	Intergenic variant	*TMEM242*	0.817
14	8115248	rs787661270	Intergenic variant	*STC1*	0.765
6	27039383	rs336712846	Intergenic variant	*CDH5*	0.747

Table S6. Correlation coefficients (r^2) between pair-wise SNPs within the 1 Mb region on SSC3 with the most significant *Fst* value.

	rs81286186	rs81372619	rs81372670	rs81372698	rs335884394	rs337169235	rs345870305	rs342302874	rs341843841
rs81286186									
rs81372619	1								
rs81372670	1	1							
rs81372698	1	1	1						
rs335884394	0.96	1	1	0.97					
rs337169235	0.96	1	1	0.97	1				
rs345870305	1	1	1	1	1	1			
rs342302874	1	1	1	1	1	1	1		
rs341843841	1	1	1	1	0.97	0.97	1	1	

Table S7. Body weight, growth-related QTLs overlapping the 1-Mb region on SSC3 with the most significant *Fst* value.

Chromosome	Starting position (bp)	Ending position (bp)	QTL_ID	Related trait
3	74291596	74437627	3891	Average feeding rate
3	79317941	79317981	139995	Feed conversion ratio
3	75917730	75917770	55829	Obesity index
3	75934381	75934421	55828	Obesity index
3	75961204	75961244	55830	Obesity index
3	76622784	119745261	894	Body weight (3 weeks)
3	81501911	81648067	9584	Daily feed intake

Table S8. Details of the fecundity related SNPs with the highest LSBL values.

Chr	Position (bp)	ID	Location	Closest gene	LSBL value
3	114228362	rs80844821	intronic variant	*NCOA1*	0.990
14	65172175	rs80838113	intronic variant	*CABCOCO1*	0.990
4	62776676	rs81302129	intronic variant	*SBSPON*	0.846
1	247929572	rs80954530	intronic variant	*ZNF462*	0.764
10	25731821	rs81422644	intronic variant	*ZNF367*	0.763
14	43736961	rs80782172	intergenic variant	*MYO18B*	0.702
10	20805520	rs81318098	intergenic variant	*LHX9*	0.683
8	41020539	rs81476904	3' UTR variant	*PDGFRA*	0.655

Table S9. Significant GO terms enriched for candidate genes under selection in seven pig breeds in the Taihu Lake region.

GO_ID	Description	Associated gene	*P* value
GO:0008283	Cell proliferation	*IGSF8*, *PELO*, *FOXC1*, *GRHL2*, *LHX9*	0.02
GO:0048015	Phosphatidylinositol-mediated signaling	*PIK3C3*, *PDGFRA*, *AGO3*	0.04
GO:0007219	Notch signaling pathway	*CNTN6*, *FOXC1*, *TMEM100*	0.04
GO:0006940	Regulation of smooth muscle contraction	*CHRNB4*, *CHRNA3*	0.04
GO:0005524	ATP binding	*QRSL1*, *MYO18B*, *HSPA2*, *TAOK1*, *PIK3C3*, *PDGFRA*, *KCNJ10*, *NSF*, *ABCA5*, *HFM1*	0.03

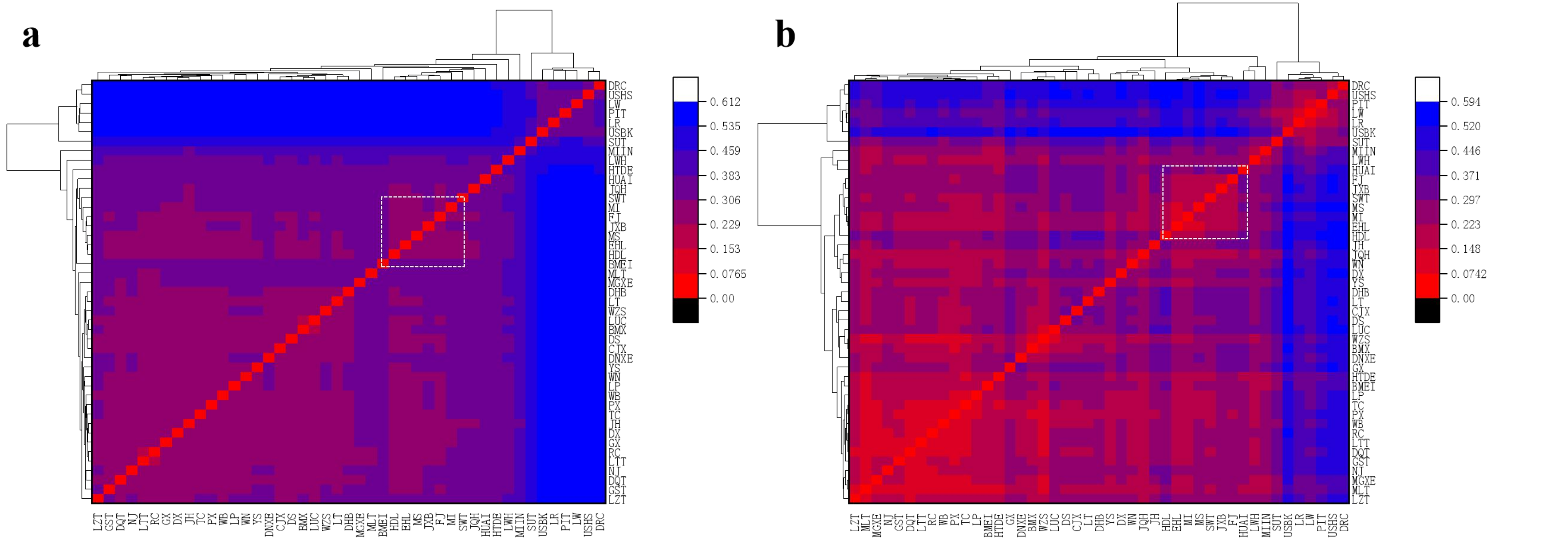

Figure S1. The heatmap reflects the genetic relationships of 1228 pigs from the 45 Eurasian breeds. (a) The heatmap that was plotted based on the genetic distance (1-Dst) matrix between pair-wise breeds. Seven indigenous pig breeds in the Taihu Lake region are highlighted in the white dotted box. (b) The heatmap that was plotted based on the genetic differentiation coefficient (*Fst*) matrix between pair-wise breeds. The seven indigenous pig breeds in the Taihu Lake region are highlighted in the white dotted box. The full name of each breed is given in Table 1.

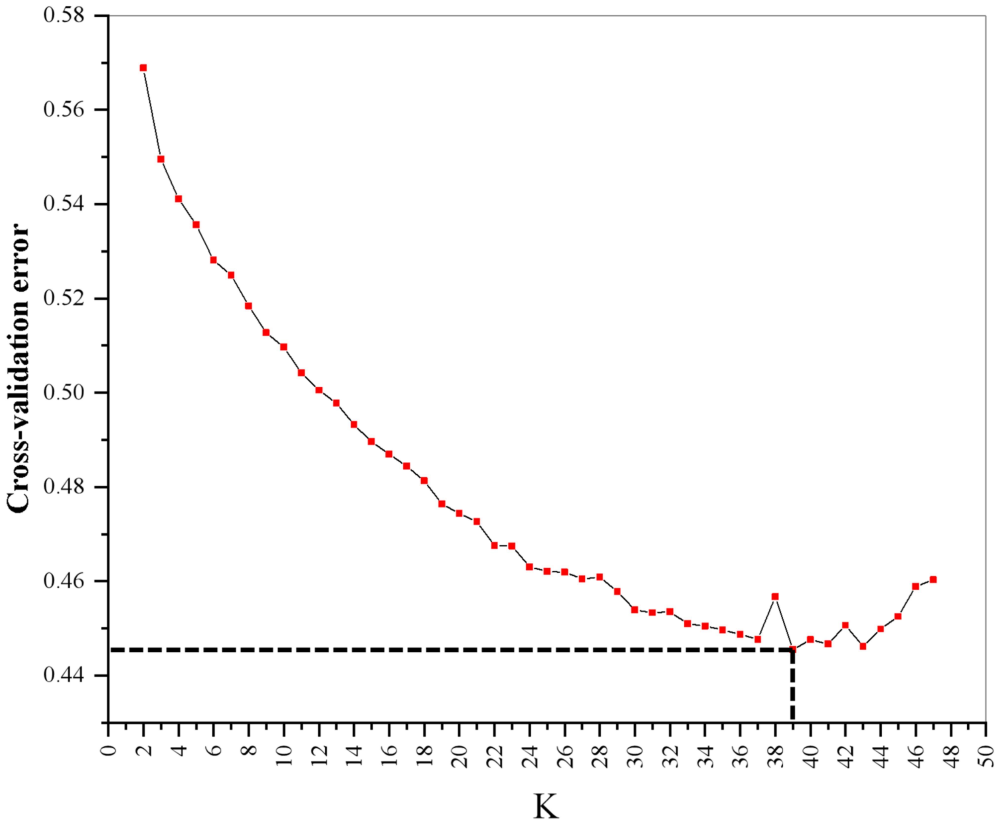

Figure S2. Cross-validation errors at different K values in the admixture analysis for the 45 Eurasian breeds.

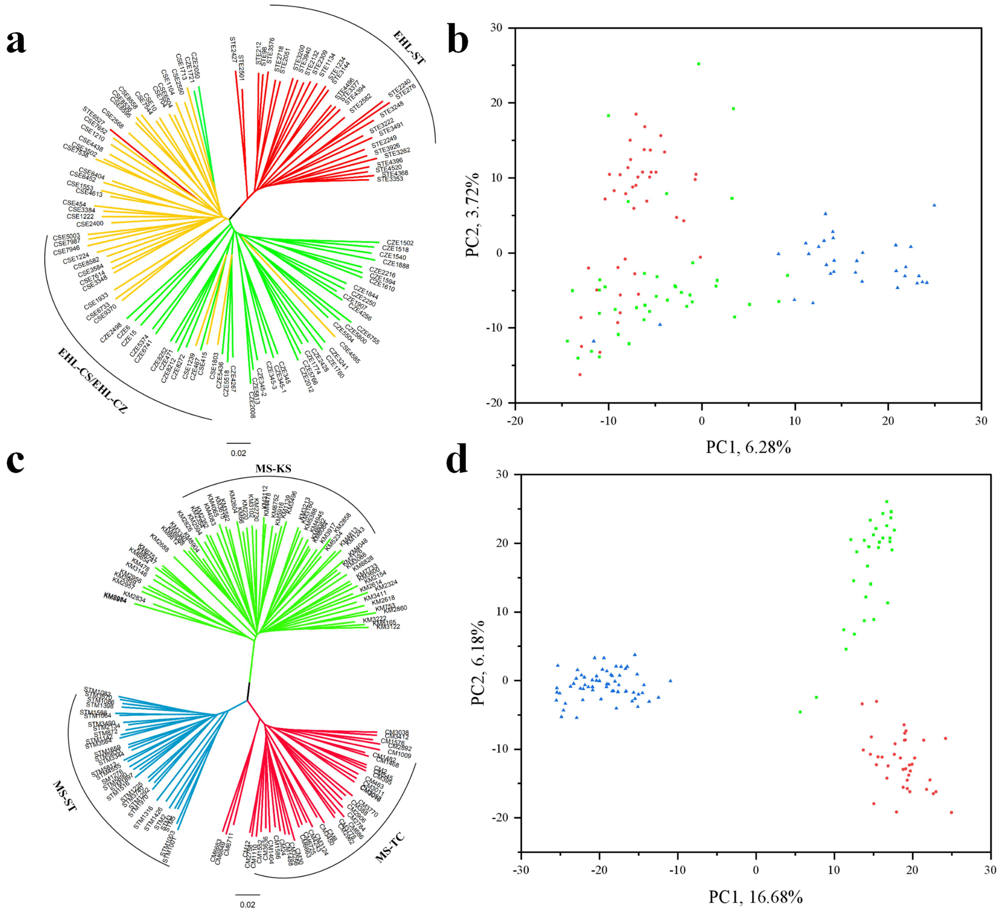

Figure S3. Genetic relatedness of Erhualian and Meishan pigs raised in multiple conservation farms. (a) Neighbor-joining tree of Erhualian pigs from three conservation farms. Individuals from the same farm are highlighted by the same color. (b) Principal component (PC) plots of Erhualian pigs from three conservation farms. The first (PC1) and second components (PC2) are shown, the percentage represents the proportion of the corresponding principal component. (c) Neighbor-joining tree of Meishan pigs from three conservation farms. Individuals from the same farm are highlighted by the same color. (d) Principal component plots of Meishan pigs from three conservation farms. PC1 and PC2 are shown, the percentage represents the proportion of the corresponding principal component.

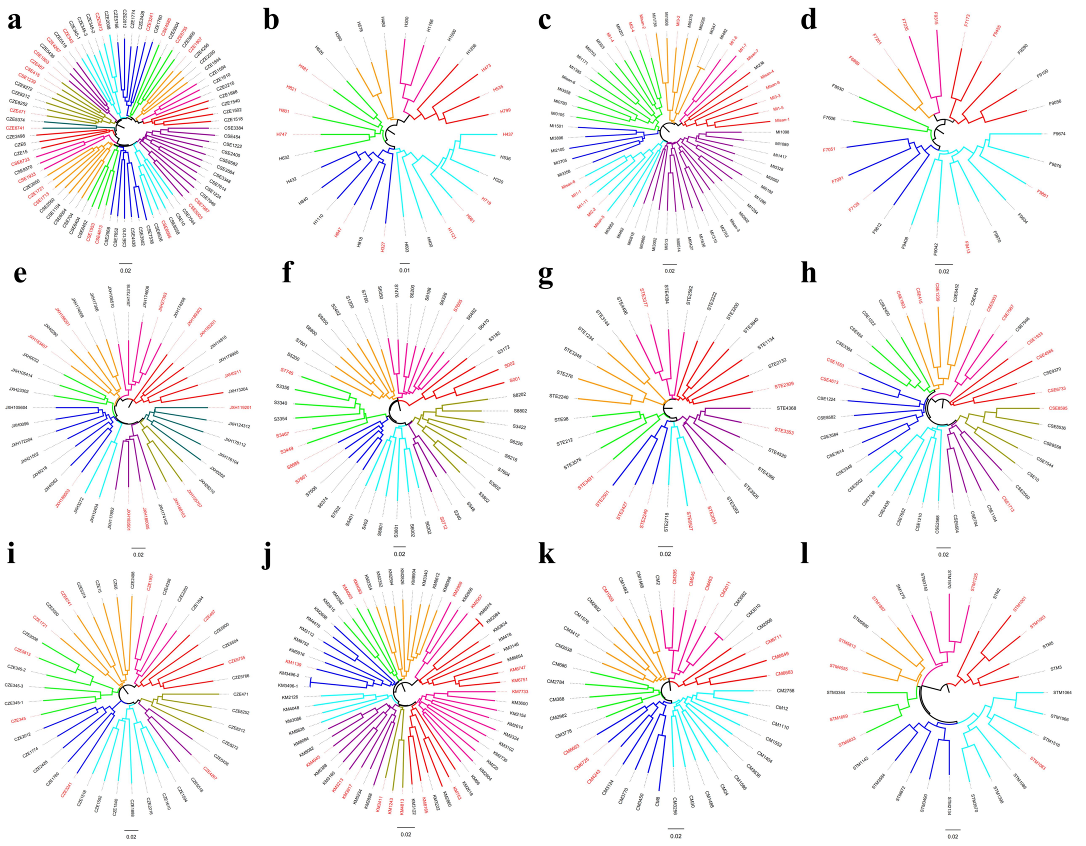

Figure S4. Population structure of all tested pigs from each conservation farm. Neighbor-joining trees were constructed based on pair-wise genetic distances (1-Dst) between individuals. Different colors represent different subfamilies. Boars are indicated with red colour. (a) Erhualian in Changzhou and Changshu (EHL-CZ/EHL-CS); (b) Hongdenglong (HDL); (c) Mi (MI); (d) Fengjing (FJ); (e) Jiaxing Black (JXB); (f) Shawutou (SWT); (g) Erhualian in Sutai (EHL-ST); (h) Erhualian in Changshu (EHL-CS); (i) Erhualian in Changzhou (EHL-CZ); (j) Meishan in Kunshan (MS-KS); (k) Meishan in Taicang (MS-TC); (l) Meishan in Sutai (MS-ST).
